# Characterization and identification of leukemia initiating cells in pediatric acute lymphoblastic leukemia

**DOI:** 10.1186/2194-7791-1-S1-A19

**Published:** 2014-09-11

**Authors:** Luca Trentin, Manon Queudeville, Sarah Mirjam Eckhoff, Nabiul Hasan, Klaus-Michael Debatin, Lueder Hinrich Meyer

**Affiliations:** Department of Pediatrics and Adolescent Medicine, University Medical Center Ulm, Ulm, Germany

In acute lymphoblastic leukemia (ALL) leukemia initiating cells (LICs) have been considered to be organized in an hierarchical fashion, however recent findings demonstrated LIC-activity also in more committed cells supporting a stochastic stem cell concept. Thus, the nature of leukemia initiating cells in ALL still remains elusive.

As an alternative approach to define LICs by expression of cellular markers as commonly employed, we addressed LIC activities in ALL by functional investigation of cellular subfractions of distinct cell cycle phases.

Patient-derived xenograft BCP-ALL cells were sorted according to cell cycle stages (i.e. G0/G1 and G2/M) and subsequently transplanted onto NOD/SCID mice. All cell fractions led to engraftment indicating LIC activity of all leukemia cells. However, cells isolated from G0/G1 cell cycle phases led to early leukemia onset in contrast to cells from late cell cycle (G2/M) constantly showing lowest LIC activity. Strikingly, this difference in LIC activity was maintained upon secondary transplantation.

In an alternative approach, we investigated metabolic activities in cellular leukemia subfractions and identified low metabolic activity in cells of early G0/G1 cell cycle phases compared to increased cellular metabolism in cells of late G2/M. To address LIC-capacities of ALL cells with distinct metabolic activities on a functional level, cellular fractions were sorted according to low or high ROS levels and subsequently transplanted onto NOD/SCID mice. Interestingly, a prolonged engraftment was observed upon transplantation of ROS^high^ cells in contrast to faster leukemia repopulation in recipients transplanted with ROS^low^ cells, showing that the metabolic activity is indicative for its leukemia initiating activity.

In summary, we identified LIC-activity in all leukemia subpopulations. Importantly, our findings indicate that leukemia initiating cells in ALL are enriched in early cell cycle and characterized by low metabolic activity.

